# Turning the tide: harnessing vaccines and viruses to fight cancer

**DOI:** 10.1093/immadv/ltaf033

**Published:** 2025-12-23

**Authors:** Callum Blee, Munitta Muthana, Greg Wells, Sarah Danson

**Affiliations:** Sheffield Ex-Vivo Group, Division of Clinical Medicine, School of Medicine and Population Health, University of Sheffield, Sheffield, United Kingdom; Sheffield Ex-Vivo Group, Division of Clinical Medicine, School of Medicine and Population Health, University of Sheffield, Sheffield, United Kingdom; Sheffield Ex-Vivo Group, Division of Clinical Medicine, School of Medicine and Population Health, University of Sheffield, Sheffield, United Kingdom; Sheffield Ex-Vivo Group, Division of Clinical Medicine, School of Medicine and Population Health, University of Sheffield, Sheffield, United Kingdom; Weston Park Cancer Centre, Sheffield Teaching Hospitals NHS Foundation Trust, Whitham Road, Sheffield, United Kingdom

**Keywords:** cancer vaccines, oncolytic viruses, pre-clinical models

## Abstract

Cancer vaccines and oncolytic viruses (OVs) represent promising immunotherapeutic strategies, harnessing adaptive and innate immune responses for targeted tumour eradication. Cancer vaccines aim to induce tumour-specific cytotoxic T lymphocytes (CTLs) through antigen presentation, while OVs mediate direct tumour lysis and stimulate immunogenic cell death, enhancing anti-tumour immunity. Despite keen interest, with over 350 clinical trials initiated since 2020, challenges persist in carrying the success seen in a pre-clinical setting to a clinical one. Advancements in preclinical modelling are essential for bridging the gap between *in vitro* findings and clinical efficacy. Traditional two-dimensional (2D) cultures, although cost-effective and reproducible, fail to recapitulate the complexity of the tumour microenvironment (TME). Three-dimensional (3D) models including spheroids, organoids, tumour-on-a-chip, and bioprinting offer improved architectural and physiological relevance, allowing for the assessment of immune cell infiltration and viral spread. *In silico* models further complement these systems by enabling high-throughput neoantigen prediction and therapy simulation. *In vivo* models such as patient-derived xenografts (PDXs), genetically engineered mouse models (GEMMs), and syngeneic models provide critical insights into tumour-immune dynamics and therapeutic efficacy in a systemic context at a whole organism level. Integrating 2D, 3D, *in silico*, and *in vivo* platforms provides a versatile basis for the preclinical evaluation of cancer vaccines and OVs. This multidisciplinary approach is vital to advancing personalized immunotherapies, improving biomarker development, and accelerating the translation of novel treatments.

## Introduction

Cancer arises due to a complex interplay of genetic mutations and epigenetic alterations [1]. These disrupt the normal cellular mechanisms governing proliferation, differentiation, and apoptosis. There were ∼20 million global incidences of cancer in 2022 and ∼9.74 million deaths, with yearly cases expected to rise to 35 million by 2050 [2]. These numbers highlight the constant need for the development of novel and more effective therapeutic interventions. The current treatment landscape is beginning to shift away from traditional treatment such as surgery, radiotherapy, chemotherapy, with research focus now being put on more specialized approaches including immunotherapy, hormone therapy, and targeted therapy.

Cancer vaccines represent a class of immunotherapy designed to elicit or enhance anti-tumour immune responses. This idea has since translated to the field of oncology in both a prophylactic and therapeutic context. Unprecedented success was observed in the prevention of certain viral related cancers such as Gardasil 9 which protects against ∼90% HPV-associated cervical cancers worldwide [3]. This was mirrored in an observational study assessing the national HPV vaccination programme in England [4]. There was a 97% reduction in the risk of grade 3 cervical intraepithelial neoplasia (CIN3) when vaccinated at ages 12–13. This reduces drastically to 34% at ages 16–18, highlighting the need for vaccination programmes at an early age. In a therapeutic context, development of cancer vaccines has faced challenges but there are anti-cancer agents approved for use in patients. Therapeutically, cancer vaccines seek to re-educate adaptive immunity by delivering defined or patient-specific antigens resulting in a tumour-specific T-cell response. Despite initial responses, therapy-resistant tumour subclones frequently persist seeding relapse. By expanding epitope breadth, therapeutic cancer vaccines provide a broader coverage of the heterogeneous clonal populations found within a tumour, as well as supporting long-term immunosurveillance.

## Mode of action

**Figure 1.**
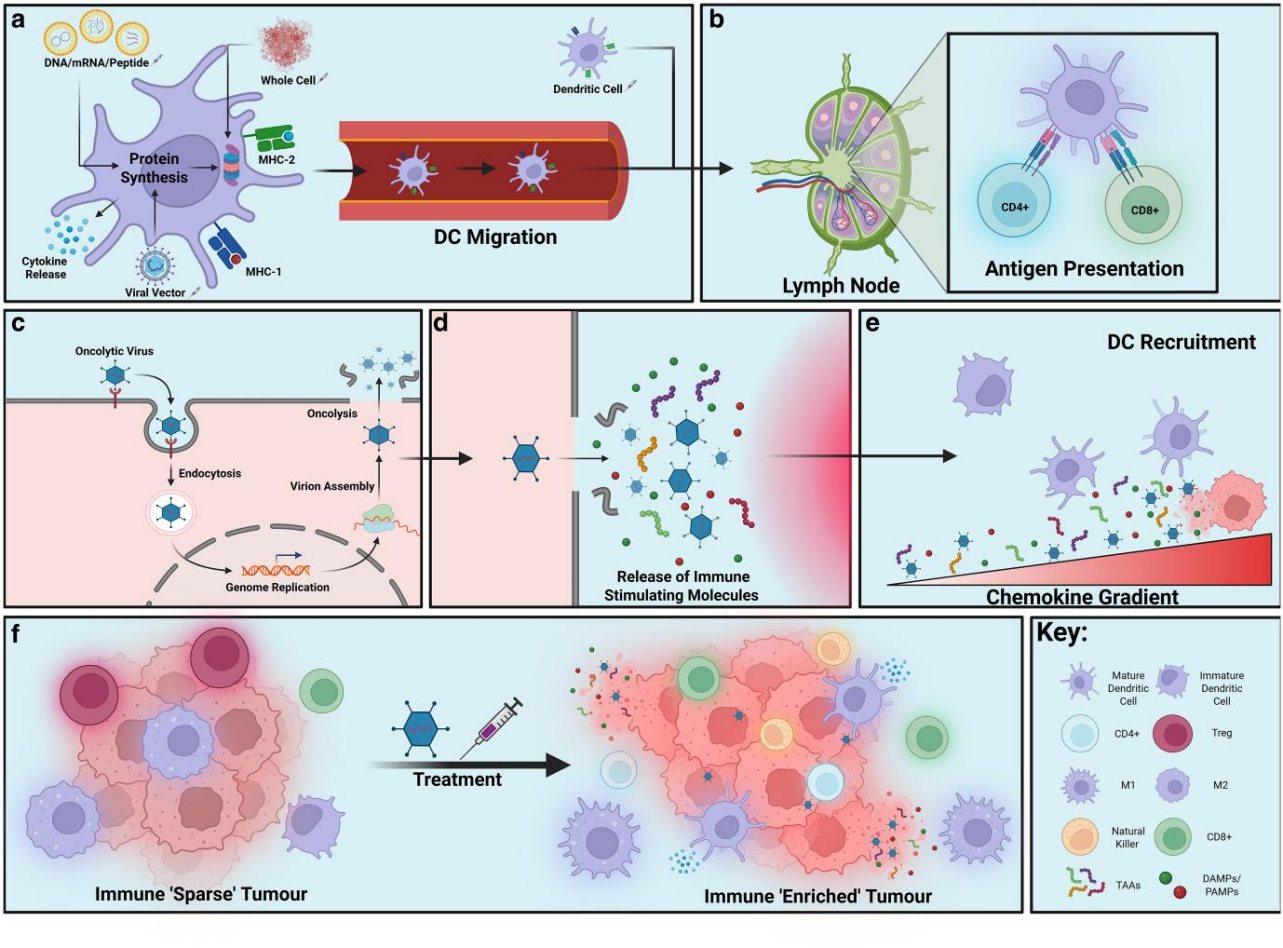
Mechanisms of action of therapeutic cancer vaccines and oncolytic viruses. (a–b) Therapeutic cancer vaccines, including DNA, mRNA, peptide, whole-cell, dendritic cell, and viral vector platforms, deliver tumour antigens that are processed by antigen-presenting cells such as DCs, leading to DC maturation, migration to lymph nodes, and activation of CD4^+^ and CD8^+^ T cells. (c–e) OVs infect tumour cells, leading to viral genome replication, oncolysis, and release of TAAs, PAMPs, and DAMPs. These immunostimulatory signals generate local inflammation and chemokine gradients that recruit DCs to the tumour microenvironment. (f) Both vaccine and OV-mediated immunomodulation contribute to the conversion of immunologically ‘cold’ tumours with low immune infiltration into ‘hot’ tumours enriched with activated immune cells, including cytotoxic T cells, natural killer (NK) cells, and pro-inflammatory macrophages, enhancing anti-tumour immunity. Created in BioRender. Blee, C. (2025) https://BioRender.com/xa7d2sc.

The mode of action of cancer vaccines fundamentally revolves around enhancing the adaptive immune response against tumour antigens (Fig. 1a and b). These vaccines aim to utilize proteins called tumour associated antigens (TAAs) and tumour specific antigens (TSAs). TAAs are expressed on normal cells but at a much lower level whereas TSAs are solely found on tumour cells as they arise from single-nucleotide variants (SNVs), frameshift, and gene fusion events [5]. After vaccine delivery, antigen presenting cells (APCs) such as dendritic cells (DCs) internalize and process these antigens by digesting them into smaller peptides. These DCs mature and then load peptides onto Major Histocompatibility Complex (MHC) molecules on their surface. DCs migrate to lymph nodes, and interact with naive CD8+ T cells (MHC class I) and CD4+ T cells (MHC class II) that have T cell receptors (TCRs) capable of recognizing the presented peptide-MHC complexes [6]. This interaction, along with co-stimulatory signals provided by the DCs, leads to the activation and proliferation of antigen-specific T cells [7]. Activated CD8+ T cells differentiate into CTLs that can directly kill cancer cells displaying the same tumor-associated peptides on their MHC class I molecules [8], initiating their destruction through perforin and granzyme release. Activated CD4+ T cells have a range of roles; they can directly activate CD8+ CTLs through interleukin-2 (IL-2) secretion, CD40 signalling to B cells, and mediating direct anti-tumour activity [9].

## Oncolytic Viruses

A second class of immunotherapies is OVs, these display *in-situ* vaccinal activity due to an ability to trigger innate immune sensors and release of a broad antigen repertoire. Mechanistically, they work differently meaning they are complementary to but distinct from therapeutic cancer vaccines. Usually termed ‘virotherapy’, they can be naturally occurring or genetically modified and selectively replicate within tumour cells, causing their subsequent lysis [10]. OVs have a unique characteristic by offering a two-pronged approach through oncolysis and activation of anti-tumour immunity (Fig. 1c–e). Mutations driving cancer cell survival often concurrently render them susceptible to OVs through the downregulation of critical antiviral innate signaling pathways [11, 12]. OVs extensively replicate within the cancer cell leading to oncolysis, directly killing the tumour cell. This process releases viral progeny, allowing for the infection of neighboring cancer cells, propagating across the tumour. The lysis of cancer cells by OVs also releases a range of TAAs and danger-associated molecular patterns (DAMPs) into the TME. These molecules act as potent immunostimulatory signals [13]. TAAs are taken up by APCs, DAMPs released during oncolysis further enhance the maturation and activation of these APCs, leading to a more robust priming of anti-tumour T cell responses [14]. Furthermore, the local inflammation induced by viral infection and cancer cell lysis can also attract other immune effector cells, such as NK cells and macrophages, contributing to tumour destruction whilst sparing healthy cells [15].

## Vaccine designs and clinical landscape

The core immunogenic principle of cancer vaccines is adaptable to various design platforms, thereby broadening therapeutic potential. These are nucleic acid; DNA (plasmid based) and RNA (mRNA), peptide, viral, bacterial, and cell-based (Table 1).

**Table 1.** Summary of key features, advantages, and limitations of major cancer vaccine designs, providing a comparative framework for optimizing vaccine-based immunotherapies.

Vaccine type	Basic composition	Advantages	Disadvantages
Peptide/Protein-based vaccines	Synthetic peptides (short tumour epitopes) or full-length recombinant tumour-associated antigens; usually administered with adjuvants or delivery systems (e.g. emulsions, nanoparticles).	Chemically defined and easy to manufacture. Safe and well-tolerated. Scalable. Compatible with multi-epitope formulations. Minimal risk of off-target toxicity.	Often poorly immunogenic. HLA-restricted (especially for peptides). Require adjuvants for efficacy. May induce tolerance. Susceptible to tumour immune escape.
Whole Tumour cell vaccines	Irradiated autologous or allogeneic tumour cells, often modified to secrete immune stimulants (e.g. GM-CSF).	Broad antigen repertoire, including unknown epitopes. Potentially captures patient-specific mutations. Avoids need for antigen identification.	Weak immunogenicity without modification. Manufacturing and standardization challenges. Potential to include immunosuppressive elements. Requires tumour cell availability.
Dendritic cell vaccines	Autologous DCs derived from peripheral blood, matured and pulsed *ex vivo* with peptides, proteins, tumour lysates, or nucleic acids; re-administered to patients.	Potent antigen-presenting capability. Induces both CD4⁺ and CD8⁺ T-cell responses. Personalized and adaptable platform.	Complex, costly, and labour-intensive manufacturing. Limited standardization. Variable DC quality between patients. Logistics and cell handling requirements.
DNA vaccines	Plasmid DNA encoding tumour antigens, delivered via electroporation, gene gun, or nanoparticles.	Stable and easy to produce. Capable of encoding multiple antigens and immune modulators. Stimulates both humoral and cellular immunity. Inexpensive and scalable.	Poor transfection and expression efficiency in humans. Weak immunogenicity. Requires an effective delivery method.
mRNA vaccines	*In vitro*-transcribed mRNA encoding tumour antigens, often chemically modified and formulated in lipid nanoparticles (LNPs) or other carriers.	Rapid and flexible manufacturing. No risk of genomic integration. Strong immune activation via innate sensing. Suitable for personalization.	Requires sophisticated delivery systems. Thermolabile; cold chain logistics needed. May induce systemic inflammation. Transient antigen expression.
Viral vector vaccines	Replication-competent or replication-deficient viral vectors (e.g. adenovirus, vaccinia, VSV) engineered to express tumour antigens.	High-level antigen expression. Strong induction of cellular immunity. Efficient delivery to antigen-presenting cells. Some vectors allow repeat dosing.	Pre-existing immunity may reduce efficacy. Anti-vector immunity limits repeat use. Safety concerns (e.g. inflammation, vector persistence). Regulatory complexity.
Bacterial vector vaccines	Live-attenuated or engineered bacteria (e.g. Listeria monocytogenes, Salmonella, BCG) delivering tumour antigens or modulating the tumour microenvironment.	Natural adjuvanticity and strong innate immune activation. Can target immune-suppressive tumour niches. Induces both humoral and cellular immunity. Suitable for mucosal administration.	Risk of systemic infection, especially in immunocompromized patients. Pre-existing immunity may neutralize effect (species-dependent). Regulatory and safety hurdles. May require antibiotic-resistance management.

DNA and mRNA vaccines deliver genetic information encoding these tumour antigens directly to APCs. DNA vaccines will undergo transcription to synthesize mRNA which is translated into the specific tumour antigen peptide by ribosomal machinery. These peptides undergo intracellular degradation and binding to MHC Class I or II before presentation to CD4^+^ or CD8^+^ T cells [16]. A phase I/II trial for patients with hepatocellular carcinoma (HCC) used a DNA plasmid (GNOS-PV02) with interleukin-12 (cytokine adjuvant) alongside pembrolizumab. An objective response rate (ORR) of 30.6% was achieved compared with 18.3% seen in a phase III trial of pembrolizumab alone [17, 18]. There was also an 86.4% neoantigen-specific T cell response across all evaluable patients. The KEYNOTE-942 phase 2b study looked at the use of an mRNA vaccine (mRNA-4157) in combination with pembrolizumab in resected melanoma [19]. Higher recurrence-free survival was observed in the combination cohort (hazard ratio [HR] for recurrence or death, 0.561 [95% Confidence Interval (CI) 0.309–1.017]), with lower recurrence or death event rate (24 [22%] of 107 vs 20 [40%] of 50). After 18 months, recurrence-free survival was higher in combination, 79% (95% CI 69.0–85.6), versus 62% (46.9–74.3) for pembrolizumab alone.

**Figure 2.**
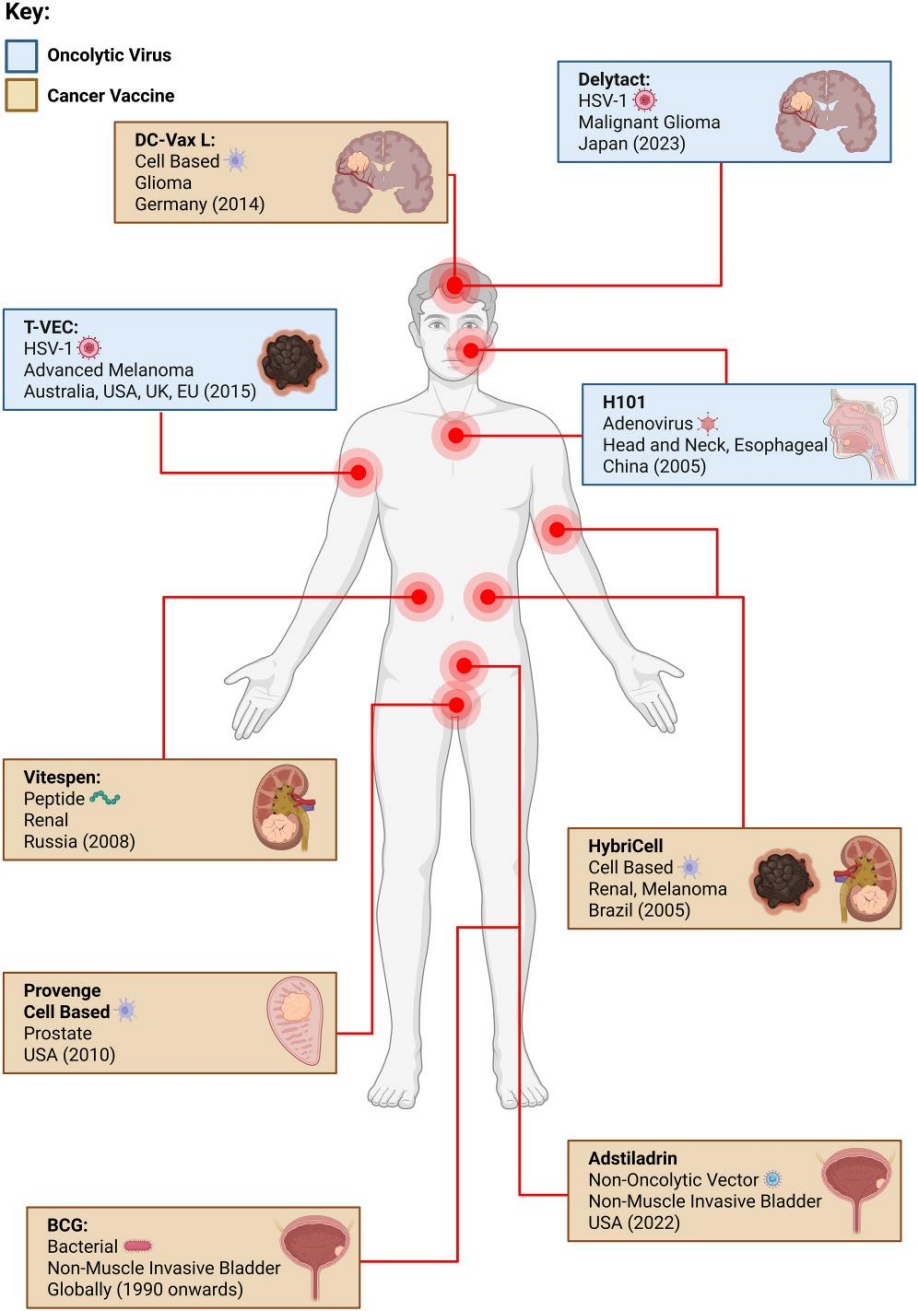
Current approval landscape of cancer vaccines and oncolytic viruses. Approved therapeutic cancer vaccines and OVs are shown with their target cancer indication, and region and year of regulatory approval. Created in BioRender. Blee, C. (2025) https://BioRender.com/o6qun3n.

Peptide vaccines utilize short and long synthetic peptides that are derived from TAAs or TSAs. These peptides are then taken up by APCs which present the peptide on MHC class I and II molecules to activate CTLs [20]. These vaccines normally carry multiple epitopes covering a range of targets to elicit a stronger immune response. Oncophage (Vitespen) is a heat-shock protein (HSP) 96 peptide vaccine and was approved in Russia in 2008 for renal cancer (Fig. 2). A phase 3 clinical trial saw no difference in recurrence free-survival with patients given this vaccine [21]. A Phase IIa study with SurVaxM, a synthetic survivin vaccine, was used to treat newly diagnosed GBM (nGBM). Progression free survival (PFS) was 95.2% (95% CI, 86.0–98.4) at 6 months in combination with the standard temozolomide course [22]. Standard temozolomide chemoradiation course has a PFS of 54% (95% CI, 0.45–0.64) at 6 months [23].

Cell-based vaccines involve tumour cells or APCs such as DCs that have been primed to enhance immunogenicity. They are primed with tumour antigens (peptides, tumour lysate), and then re-infused back into the patient [24]. Tumour cell vaccines are either derived from the patient's own tumour (autologous) or from another individual or cancer cell line (allogeneic) [25]. Cell-based vaccines tend to present a broader range of antigens to the immune system, again, eliciting a greater immune response while limiting the issue of tumour heterogeneity. Provenge (Sipuleucel-T) is an FDA approved cell-based immunotherapy where the patient's own immune cells, mainly DCs, are primed with prostatic acid phosphatase (PAP) [26]. The PAP antigen is expressed in over 95% of primary prostate adenocarcinomas, therefore making it a rational target for anti-cancer therapy [27]. The IMPACT trial in which 314 patients received Sipuleucel-T, there was a relative reduction of 22% in the risk of death compared with the placebo group ([HR], 0.78; [95% CI, 0.61–0.98; *P* = .03]). This resulted in a 4.1-month improvement in median survival (25.8 vs. 21.7) [28]. HybriCell, a DC fusion vaccine approved in Brazil in 2005 was evaluated in 35 patients either with renal cell carcinoma (RCC) or melanoma. 71% showed stable disease [29]. DCVax-L, a DC vaccine against GBM, has been approved for compassionate use in Germany. A recent phase 3 clinical trial showed that addition of DCVax-L to standard of care (SOC) treatment extended median overall survival (OS) in both *n*- and rGBM [30]. Median OS for nGBM receiving DCVax-L was 19.3 (95% CI, 17.5–21.3) months vs 16.5 (95% CI, 16.0–17.5) in control patients ([HR] = 0.80; [98% CI, 0.00–0.94; *P* = .002]). For rGBM median OS was 13.2 (95% CI, 9.7–16.8) months from relapse vs 7.8 (95% CI, 7.2–8.2) months among control patients ([HR], 0.58; [98% CI, 0.00–0.76; *P* < .001]).

Bacterial-derived cancer vaccines harness the ability of bacteria or their components to stimulate anti-tumour immune responses. These approaches can involve using live attenuated bacteria to directly target tumours and elicit local immunity. Bacillus Calmette-Guérin (BCG), which is conventionally employed for tuberculosis prophylaxis, has proven effective in reducing recurrence and progression of high risk non muscle-invasive bladder cancer (NMIBC). A meta-analysis review of 9 randomized trials showed a complete response (CR) rate of 68.1% [31]. Bacterial ghost vaccines are an alternative where an empty bacterial shell can be loaded with tumour antigens. The original surface PAMPs remain to elicit an immune response [32]. To date, only pre-clinical studies have been completed, one of which used cancer cell membrane-coated liposomal paclitaxel-loaded bacterial ghosts to treat metastatic lung cancer in mice. Treatment showed an increase in immune activity with a relatively high safety profile observed across major organs [33].

Viral vector vaccines utilize modified viruses to infiltrate immune cells delivering tumour antigens. This delivery prompts the host's immune system to recognize and mount a targeted response against cancer cells. Clinical trials have faced disappointing outcomes in some instances, often failing to meet initial expectations. A Phase I trial involving ChAd68 and self-amplifying mRNA in combination with ipilimumab and nivolumab observed disease progression in 15/19 patients [34]. To date there are no approved nonreplicating viral vector vaccines. Nadofaragene Firadenovec (Adstiladrin), a nononcolytic Adenovirus vector based gene therapy is used in the treatment of high-grade, BCG unresponsive NMIBC. The adenoviral vector carries the interferon alpha-2b (Intron A) gene which is then expressed in cells within the bladder wall [35]. Intron A is a cytokine resulting in immune system activation. It was granted FDA approval in 2022 [36]. A phase III clinical trial saw a CR of 53% after 3 months [37].

## OV landscape

Virotherapy relies on oncolytic viral vectors which are often modified to enhance their tumour selectivity, increase their oncolytic potency, reduce off-target effects, and enable the delivery of therapeutic transgenes to further augment anti-cancer activity [38]. There are currently three approved OVs; Talimogene Laherparepvec (T-VEC), Teserpaturev (Delytact), and Oncorine (H101) (Fig. 2). Approved by the FDA, NICE, and across most of Europe, Talimogene T-VEC is a modified Herpes Simplex Virus 1 (HSV-1) used to treat advanced melanoma [39, 40]. Success across other cancers has been varied with positive results for triple negative breast cancer (TNBC) [41], but limited drug efficacy in those with HCC [42]. Another approved HSV-1 treatment, Delytact, was approved in Japan in 2023 for malignant glioma. A phase II study with a 1 year survival rate of 84.2% led to its approval [43]. H101 is the only adenoviral derived OV and solely approved in China. It was approved for head and neck and oesophageal cancer, but a recent pilot study in combination with nivolumab, shows it has therapeutic potential in HCC [44].

## Clinical landscape

**Figure 3.**
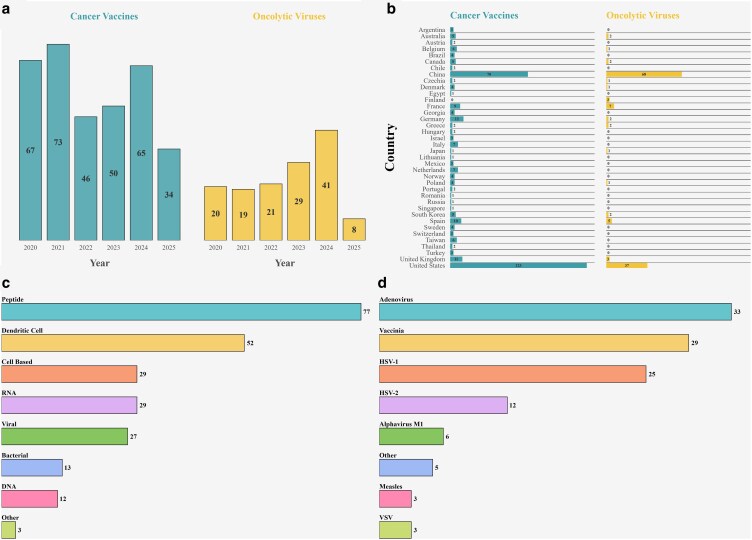
Current clinical landscape of cancer vaccines and oncolytic viruses. (a) Annual counts of therapeutic cancer vaccine and OV clinical trials registered from 2020 to 2025. (b) Geographic distribution of trials by country. (c) Breakdown of cancer vaccine platforms in active clinical studies. (d) Distribution of OV platforms in trials. *As of June 2025 (Several trials are multi-location hence the disparity in counts).

From January 2020 to April 2025, there have been 242 trials registered under the keywords ‘Therapeutic Cancer Vaccines’ and ‘Cancer’ on the United State’s National Library of Medicine ClinicalTrials.gov website (Fig. 3). Peptide and DC vaccines account for over 50% of these trials. The keywords ‘Oncolytic Virus’ and ‘Cancer’ brought up 113 studies beginning from January 2020 testing numerous different OVs. Both searches showed these therapeutics are being trialled against a wide range of solid cancers, quite often in combination with pre-existing therapies.

## Challenges in cancer vaccine development

Developing effective cancer vaccines and OVs faces a complex array of challenges that span from early stage research to clinical translation.

Cancers exhibit significant variability both between patients and within the same tumour, making it difficult to identify universal TSAs that can be targeted by a single vaccine. This presence of heterogeneous cell populations will also vary susceptibility to OV infection [45]. Tumours often create an immunosuppressive microenvironment that actively suppresses immune cell activity, which can hinder the ability of vaccine-induced immune responses to effectively infiltrate and eliminate cancer cells. This involves various mechanisms, including the recruitment of immunosuppressive cells and the expression of inhibitory molecules [46, 47]. Many TAAs are self-antigens or only weakly immunogenic, meaning they don't readily trigger a strong and durable immune response. Linked to that, selecting the most appropriate antigens that are truly tumour-specific is particularly challenging for personalized vaccines where neoantigens derived from individual tumour mutations are targeted.

Identifying reliable biomarkers to predict which patients will respond to a specific vaccine and to monitor the efficacy of the treatment during clinical trials is crucial but often lacking. Similarly, biomarkers that would help identify patients that are likely to benefit from OV therapy are few and far between. Personalized neoantigen vaccines, while promising, are often expensive and complex to manufacture on a large scale, posing logistical challenges for widespread clinical application [48]. Strategies to protect the virus during transit and enhance tumor-specific targeting are crucial, these could include encapsulation within nanoparticles [49]. Running alongside this, pre-existing or rapidly induced antiviral immune responses can prematurely clear the OV, limiting its therapeutic efficacy. Although a study looking into Newcastle Disease Virus saw prior immunity enhanced anti-tumour effects *in vivo* [50].

## Models

Preclinical cancer models play a critical role in understanding cancer biology, evaluating the efficacy and safety of novel therapies ultimately guiding clinical trial design. The choice of an appropriate model is crucial for generating translatable results (Table 2). Traditionally, cancer research has relied heavily on 2D established commercial cell cultures and animal models. However, these models have limitations in recapitulating the complex *in vivo* TME human physiology, intra/inter patient tumour heterogeneity, often leading to discrepancies between preclinical findings and clinical outcomes. In recent years, there has been a growing emphasis on developing and utilizing more sophisticated 3D cell culture models and *in silico* (computational) models to better mimic the intricacies of human cancer.

**Table 2.** Summary of key models involved in anti-cancer research, and direct application to cancer vaccine and oncolytic virus development.

Model type	Key features	Applications	Limitations
2D Models	Simple. Cost-effective. Highly reproducible. High-throughput screening.	Screening vaccine components (peptides, proteins, mRNA). Basic immune co-culture assays.	Lack of tumour heterogeneity. No ECM architecture. Cannot provide nutrient/oxygen gradients.
Spheroids (3D)	Multicellular aggregates. Replicate gradients and intercellular signalling.	Assessing viral spread. CTL infiltration. Tumour-immune cell interactions in a pseudo-3D environment.	Variability in shape/size; Limited stroma and vasculature. Not fully representative of tumour complexity.
Organoids (3D)	Derived from patient tumours or stem cells. Retain key genetic and phenotypic patient features. Require autologous peripheral blood mononuclear cells (PBMCs).	Testing personalized vaccines. Patient-specific neoantigen profiling, immune interactions.	Time-intensive generation. Specialized growth conditions. Incomplete TME and immune mimicry.
Tumour-on-a-chip (3D)	Integrates microfluidics and 3D cell culture. Allows precise environmental control.	Simulates T cell migration. Real-time immune surveillance under dynamic conditions.	Limited spatial fidelity. Lacks systemic immunity.
Bioprinting (3D)	Precisely places cells, ECM components, and vasculature in predefined architecture.	Investigating vaccine/OV penetration. CTL infiltration. Tumour mechanics.	Expensive. Immature vasculature. Issues in long-term tissue viability and immune integration.
*In Silico* Models	Computational algorithms and multi-omics integration. Model immune responses and tumour behaviour.	Neoantigen prediction. Therapy simulation. Patient-specific vaccine design. Target discovery.	Heavily reliant on input data accuracy. Experimental validation required. May miss nonlinear interactions.
PDX Models	Patient-derived tumour implants in immunodeficient/humanized mice. Retain tumour diversity.	Evaluate personalized vaccines/OVs. Study tumour-specific immune responses *in vivo*.	Immune limitations in nonhumanized models. Expensive and low throughput.
GEMMs	Mice engineered with human-like mutations. Develop spontaneous tumours with intact immune systems.	Study tumour evolution and metastasis. Development of vaccines for genetically defined cancers.	Species-specific differences. Incomplete human TME mimicry. Expensive.
Syngeneic	Immunocompetent. Hosts preserve intact innate/adaptive immunity. Fast, scalable, and low-cost with reproducible tumour take.	Demonstrate efficacy and immune memory. Optimize dose, route, and timing for vaccines/OVs. Test combination therapies.	Fails to replicate structural phenotype of human tumours. Limited diversity, lack of murine cell lines. Murine and human immunity differ.

## 2D

Virtually all anti-cancer drugs are first trialled in 2D models. *In vitro* 2D methods are a valuable tool in early stage research due to their simplicity, cost-effectiveness, and easy reproducibility [51]. In the context of cancer vaccine development, which require an immune complement, they are typically employed to look at T-cell activation, recognition, and killing at a basic level in straightforward tumour-immune co-cultures [52, 53]. In the context of DC vaccines, 2D co-cultures have been done to confirm successful priming before escalation to *in vivo* mouse studies [54]. The same platforms enable rapid, mechanistic combination therapy screens to triage synergies prior to *in vivo* studies [55]. This model fails to recapitulate the intricate and dynamic physiological properties of the *in vivo* TME, including crucial aspects like heterogeneous cell-cell interactions [56]. The formation of critical nutrient and oxygen gradients, and the influence of the extracellular matrix (ECM) architecture is also missing while the freedom to access nutrients throughout does not mirror that expected *in vivo*.

## 3D

The recent and ongoing advancements in 3D cell culturing techniques offer a significant leap forward in preclinical modelling, providing a more biologically relevant platform for evaluating cancer vaccines and OVs. These models include spheroids, organoids, tumour-on-a-chip, and bioprinting.

Spheroids are the result of spontaneous aggregation of cancerous cells and integrin ECM interaction, resulting in intercellular E-cadherin interaction [57]. This architecture promotes cell-cell communication and can induce phenotypic changes associated with drug resistance and altered metabolism, more closely resembling the *in vivo* situation [58]. As such, spheroids are well suited for the study of the spatial dynamics of viral entry and are a great template for mapping spread and oncolysis [59]. These platforms have also been used to benchmark specific oncolytic constructs, directly measuring replication, spread, and therapeutic effect of the vaccinia virus TG6002 [60]. They provide a more physiologically relevant platform to validate vaccine-primed T-cell function by quantifying infiltration and tumour cell killing [61]. More broadly they can provide insights into the physical barriers and cellular interactions that might occur *in vivo* [62]. The main limitation of spheroids is that they often exhibit variability in size and shape, affecting reproducibility. They also fail to fully recapitulate the complex stromal components and vascularization of a real tumour [63].

Organoids are predominantly derived from patient tumour tissue or pluripotent stem cells, enabling them to more accurately reflect the unique genetic, epigenetic, and phenotypic characteristics of an individual's cancer [64]. As organoids retain patient-specific architecture and genomic context, this enables validation of neoantigen targets and functional assessment of vaccine-primed effectors with immune co-culture [65, 66]. A recent study used patient-derived breast cancer organoids to engineer new oncolytic viral vectors with more clinical relevance for *in vivo* use [67]. Compared with spheroids, organoids are complex and time-consuming to culture, often requiring specialized growth factors and conditions [68]. Organoid co-cultures that preserve MHC-restricted T-cell recognition generally rely on autologous PBMCs, a dependency that lowers throughput and complicates standardization. They also fail to fully recapitulate the native tumour and immune microenvironment.

Tumour-on-a-chip combines the principles of microfluidics and 3D cell culture. This involves the precise manipulation of fluids at a microscale, allowing researchers to control the environment surrounding tumour cells with high accuracy [69]. This enables the simulation of crucial physiological factors like blood flow, immune cell trafficking, controlled nutrient flow, oxygen gradients, and waste removal. A bladder cancer-on-a-chip that incorporates stromal, myeloid, and endothelial elements under microfluidic flow recapitulate key features of BCG immunotherapy, reducing tumour viability and elevating pro-inflammatory cytokines [70]. Alternatively, these platforms have also been used to track tumour engagement of primed DCs, and controlled microfluidic flow, permitting live observation of vaccine-activated T-cells toward 3D tumour spheroids [71, 72]. Extending beyond vaccines, the microfluidic chip system has been utilized to explore three tumour killing pathways of the OV, AD4-GHPE [73]. These models are limited heavily by an inability to accurately reproduce the 3D structure of the tumour niche, and will not fully capture the systemic immune responses elicited by these therapies *in vivo* [74].

Bioprinting is a technique that enables the precise reconstruction of the three-dimensional architecture of tumours with a high degree of control over the spatial arrangement of cancer cells, stromal cells, and the ECM [75]. This level of structural complexity is crucial for studying how the TME can influence the penetration and distribution of vaccines, OVs and the subsequent infiltration of activated immune cells. One study developed a long-term 3D bioprinted colorectal cancer model for screening advanced therapeutics such as OVs, allowing for the evaluation of penetration kinetics [76]. Bioprinting is still a relatively nascent technology, it lacks the ability to produce a well-established vascular model, the process is very costly, and there are still questions over the long-term viability and functionality [77].

3D models encompass a growing array of sophisticated models all aimed at bridging the gap between simplistic 2D cultures and complex *in vivo* environments. These 3D models provide a crucial platform to directly assess vaccine-induced immune cell infiltration, target cell recognition within a complex 3D structure, and cytotoxic killing under more physiologically relevant conditions. By yielding more predictive preclinical data on vaccine and viral efficacy, assessing mechanisms of action within a more human-relevant context, these advanced 3D models hold the potential to accelerate the translation of novel therapies.

## Computational

*In silico* models offer a powerful and complementary approach to experimental models, providing an unbiased, often high-throughput, and integrative perspective on cancer biology and therapeutic development [78]. These methods are beginning to bridge the vaccine and virus development pipeline end-to-end. At the discovery stage, *in silico* models can be used to identify novel TAAs and TSAs with high immunogenic potential based on genomic, transcriptomic, and proteomic data analysis [78]. Recent frameworks further refine this step by integrating proteogenomics and immunopeptidomics to improve the identification of clinically relevant antigenic peptides [79]. Following this, they can be used for epitope prediction to establish which peptide fragments derived from tumour antigens are most likely to be presented by HLA molecules and recognized by T cells. A meta-analysis study of 94 clinical trials presented a model to predict cancer vaccine immune response based on the six Human Leukocyte Antigen (HLA) class I alleles [80]. Building on the same machinery, by analysing individual patient tumour sequencing data, these tools can identify patient-specific neoantigens and predict their immunogenicity, guiding the design of truly tailored vaccines [81].

Upstream of this, computational approaches are increasingly integrated into construct and design optimization, accelerating development by rationalizing peptide assembly, adjuvanting, and manufacturability workflows [82, 83]. Clinically, such prediction-driven pipelines have enabled individualized mRNA and neoantigen vaccines that elicit tumour-specific T-cell responses in patients [84]. Additionally, an *in silico* T-VEC study established individualized immuno- and virotherapy combinations, which successfully recapitulated existing trial results [85]. Ultimately, the combination of diverse ‘omics’ datasets encompassing genomics, transcriptomics, and proteomics with clinical data is enabling the identification of critical predictive biomarkers, individualized vaccines, and patient response, facilitating more successful clinical trials.

## In vivo studies

Cancer mouse models provide a living system to study tumour biology, progression, and response to therapy. They allow researchers to investigate cancer development in a complex biological environment that more closely mimics human disease than *in vitro* studies. Mouse models include PDXs, humanized PDXs, GEMMs, and syngeneic tumour models.

Classical PDXs are established by implanting patient derived tumour tissue into an immunodeficient mouse, allowing for the engraftment and propagation of the human tumour *in vivo* [86]. As a result of their immunodeficient status, vaccine research in these models is sparse, and typically rely on humanized PDX models which are a more complete preclinical model. Humanized PDX melanoma models have shown that combining BCG with NHWD-870 elicits notable T-cell proliferation and anti-tumoural response [87]. In parallel, JAK1/2 blockade can resensitize viral-resistant tumour cells to the measles virus in GBM and ovarian cancer [88]. Another study reported that treatment with HSV-1-OX40L reinitiated tumour-infiltrating immune cells and maturation in head and neck squamous cell carcinoma models [89]. The drawbacks of humanized PDXs include loss of tumour heterogeneity, and clonal dynamics where certain cell clones outcompete others, diminishing predictive power.

GEMMs are an alternative mouse model created by introducing specific genetic alterations into the mouse germline. This leads to the spontaneous development of tumours that often recapitulate key aspects of human cancers, including their genetic drivers in the context of an intact murine immune system [90]. Model wise GEMMS have limited use for studying these types of immunotherapies, they have been used to study a modified oncolytic myxoma virus which showed high efficacy for small-cell lung cancer. Also observed were prolonged host immune cell infiltration and an increase of OS post-treatment [91]. GEMMs as a model have limitations, the murine TME lacks the specific cellular and molecular components you would expect to see in the relevant human TME. There are inherent differences between human and murine immune profiles and responses, and these models are costly and time consuming to develop [90].

## Syngeneic

Syngeneic tumour models remain the most widely used platforms for cancer-vaccine studies. The principal involves implanting a mouse tumour cell line into genetically identical immunocompetent mice [92]. Retaining intact antigen presentation and antiviral pathways, these models enable rapid, mechanistic optimization of vaccine platforms and OVs. Foundational mRNA platforms showed that DC-targeting RNA-Lipoplex vaccines induce strong effector and memory T-cell responses and induction of IFN-α release [93]. More recently, lipopolyplex mRNA neoantigen vaccines generated robust CD8⁺ responses that suppressed CT26, MC38, and B16F10 tumours, conferred memory on re-challenge, and further improved with checkpoint blockade [94]. Beyond nucleic-acid platforms, heterologous Maraba MG1 prime-boost regimens consistently expand tumour-specific CD8⁺ T cells dramatically extending median OS [95]. A further study using an adenovirus vector alongside cytokine-armed MG1 boosted MG1 vaccine tumour-specific response in various murine cancer models [96].

Syngeneic systems can also capture both direct oncolysis and vaccine-like cross-priming. Intratumoural NDV converts ‘cold’ B16 tumours into inflamed lesions, mediates control of distant metastatic sites, and synergizes with the immune checkpoint inhibitor anti-CTLA-4 [97]. In the GL261 GBM cell line, a single intracranial dose of HSV-1 prolonged survival in all treated mice. Subsequent reintroduction of parental GL261 cells was rejected on re-challenge, indicating the development of adaptive anti-tumour immunity [98]. mJX-594, a targeted GM-CSF-armed vaccinia virus, remodelled the murine TME, driving T-cell tumour infiltration and upregulation of immune-related gene signatures such as DC maturation and type-1 IFN signalling [99]. Despite extensive use, syngeneic models have well-recognized limitations. Their exclusively murine immune system and TME limit translational fidelity, and they cannot fully reproduce the cellular diversity, spatial architecture, and clonal heterogeneity of human tumours [100]. The number of commonly used lines is relatively small and uneven across cancer types, further narrowing biological application. These factors can lead to discrepancies between preclinical results and clinical outcomes, potentially contributing to the high failure rate of clinical trials.

## Concluding remarks

In summary, this review highlights the current landscape of cancer vaccine and OV development, underscoring the fundamental principles of these anti-cancer therapies and gaps in our knowledge. The development of effective cancer vaccines and OVs represents a shift in cancer therapeutics, towards harnessing the adaptive immune system for targeted tumour destruction. The fundamental mode of action for cancer vaccines centres on the presentation of tumour antigens by APCs. OVs, conversely, offer a dual mechanism of action through direct tumour lysis and the release of immunostimulatory signals that enhance anti-tumour immunity.

With over 350 clinical trials registered since the start of 2020, there is a clear sustained interest and investment in these anti-cancer therapies. However, significant hurdles persist. Tumour heterogeneity demands the development of more refined antigen selection strategies, utilizing patient-specific neoantigens identified through advanced sequencing technologies. Overcoming the immunosuppressive TME remains a critical focus, with combination therapies incorporating checkpoint inhibitors, cytokines, or other immunomodulatory agents showing promise in preclinical and clinical settings. Furthermore, the development of robust biomarkers to predict patient response and monitor treatment efficacy is paramount for guiding clinical application.

The evolution of preclinical modelling, from 2D cell cultures to the increasing sophistication of 3D models, offers a more physiologically relevant platform for evaluating therapeutic efficacy and elucidating mechanisms of action. The integration of *in silico* modelling, leveraging large-scale omics data for target identification and response prediction, further enhances our ability to rationally design and optimize these therapies. While imperfect, *in vivo* models provide critical insights into tumour-immune interactions and the translational potential of novel therapeutics at an organism-level. The future of preclinical testing for cancer vaccines and OVs lies in the synergistic integration of *in vitro* (both 2D and advanced 3D models), *in vivo*, and *in silico* data. By combining the high-throughput capabilities of 2D screening with the physiological relevance of 3D models and the predictive power of computational approaches, researchers can develop a more patient-specific understanding of vaccine efficacy. *In vivo* models can then provide critical insights into tumour-immune interactions and the translational potential of novel interventions. These efforts are essential to translating the biological promise of these interventions into practical benefits for cancer patients.

## Data Availability

No new data were generated or analysed in support of this research, data sharing is not applicable.
